# An Adolescent Female with Bipolar Disorder Presenting with Lithium-Induced Hyperthyroidism

**DOI:** 10.1155/2020/1283464

**Published:** 2020-02-12

**Authors:** Pratibha Rana, Patria Alba Aponte, Ghufran Babar

**Affiliations:** ^1^Children's Mercy Hospital, 3101 Broadway Blvd., Kansas City, MO 64111, USA; ^2^Department of Pediatric Endocrinology, Children's Mercy Hospital, 3101 Broadway Blvd., Kansas City, MO 64111, USA

## Abstract

Lithium therapy has been associated with several endocrine disorders including thyroid dysfunction, diabetes insipidus, and hyperparathyroidism. While its suppressive effect on thyroid function is well known, it is very rare to observe lithium-induced hyperthyroidism especially in the pediatric population. Here, we describe a case of lithium-induced hyperthyroidism in an adolescent female with bipolar disorder. The patient is a 17-year-old female who was treated with lithium for bipolar disorder and presented with symptoms and laboratory findings consistent with hyperthyroidism. Since thyroid autoantibodies were negative, thyroid dysfunction was attributed to lithium toxicity. Indeed, her clinical and biochemical hyperthyroid state resolved after stopping lithium therapy. Lithium-associated hyperthyroidism can occur in the pediatric population. We propose close monitoring of thyroid hormone levels in children on lithium therapy.

## 1. Case Report

Bipolar disorders are common in adolescents, with a lifetime prevalence of 2.5% [1] and 0.1% in children aged 9–13 years [2]. Lithium is commonly used for the treatment of bipolar disorders in children and adolescents [3]. It has been associated with several endocrine disorders including thyroid dysfunction, diabetes insipidus, and hyperparathyroidism. Its suppressive effect on thyroid function is well known; in contrast, lithium-induced hyperthyroidism is rare with a prevalence in the range of between 1% and 1.7% in the adult population [4, 5]. The first case of thyrotoxicosis in a patient treated with lithium was reported in 1974 [6]. Hyperthyroidism in lithium-treated patients has been associated with diffuse goiter, toxic multinodular goiter, and painless thyroiditis [7]. Lithium is also implicated in granulomatous thyroiditis, and it is reported to induce auto-antibody formation and autoimmunity in susceptible individuals [8]. There have not been any cases reported about lithium-induced hyperthyroidism in pediatric population. We are presenting an adolescent girl treated with lithium who presented with hyperthyroidism.

## 2. Methods

Retrospective chart review of the case study was carried out. Since it is a single case report, an approval from the Institutional Review Board (IRB) and consent are not required. However, when a new patient is seen in our clinic, the family signed a consent form for treatment and possible use of any clinical finding to report anonymously.

## 3. Case Presentation

Our patient is a 17-year-5-month-old female with bipolar disorder who was treated with lithium. She had been placed on Concerta, Abilify, and Lamictal before being switched to lithium monotherapy due to the limited success of these initial therapies. She responded well to lithium for two years until about 3-4 weeks prior to presentation, when she developed dizziness, heat intolerance, excessive sweating, tremors, and palpitations. Due to these symptoms, she stopped taking lithium and sought medical attention at her primary care provider's (PCP's) office. The PCP ordered laboratory tests which revealed a `suppressed TSH (<0.02 mcIU/mL, normal range: 0.35–5.50) and an elevated free T4 (3.5 ng/dL, normal range: 0.8–1.9), both consistent with hyperthyroidism. Her lithium level at that time was <0.3 (normal range: 0.6–1.2 mmol/L), likely due to noncompliance during the past two weeks. The PCP received these results on the weekend, and the patient was referred to the emergency department (ED) where, on examination, she was tachycardic (140 bpm), afebrile, and her blood pressure was 132/84 mmHg. Her thyroid exam revealed the presence of a nontender goiter; no nodules were palpable. Repeat laboratory tests at the ED confirmed biochemical hyperthyroidism (TSH: <0.02 mcIU/mL, total T3: 351 ng/dl, normal range: 55 to 209) and an elevated free T4 (3.7 ng/dL). There was a prolonged QT interval on her EKG. Of note, our patient had no family history of autoimmunity or thyroid disease. Thyroid suppressive therapy was initiated with methimazole, 10 mg PO 3 times a day (0.46 mg/kg/day) as advised by the endocrinologist. She was also started on propranolol (60 mg/day∼0.92 mg/kg/day). The ESR, CRP, CBC, and BMP were normal. Thyroid autoantibodies (antithyroid peroxidase antibody, antithyroglobulin antibody, and thyroid stimulating immunoglobulin) were all negative. She was advised not to restart lithium and consult her psychiatrist. She was seen in the endocrine clinic about two weeks later, after being off-lithium for four weeks; her hyperthyroid symptoms had improved; however, she still had palpitations and heat intolerance, while her heart rate and blood pressure were still above the age appropriate ranges (101 bpm and 133/74 mm hg, respectively). She had mild tongue fasciculation but no exophthalmos. Repeat thyroid function tests showed a lower free T4 level (2.0 ng/dl) as compared with two weeks ago, while the TSH was still suppressed. A subsequent endocrine visit two weeks later showed a normalized free T4 (1.5 ng/dl) and improved clinical hyperthyroidism. As a result, propranolol was discontinued, while methimazole treatment was maintained. By three months after her initial presentation, the methimazole dose was reduced (10 mg b.i.d.) due to a borderline-low (0.8 ng/dl, normal range of 0.8 to 1.9 ng/dL), Methimazole was eventually discontinued after five months of initial presentation. At her last follow-up in the endocrine clinic (seven months after diagnosis of hyperthyroidism and several weeks after stopping methimazole), she was clinically and biochemically euthyroid (Table 1).

## 4. Discussion

The prevalence of bipolar disorders in adolescence ranges between 1.8% and 2.5% [9, 10]. They are considered as the fourth leading cause of disability among adolescent worldwide. In children with bipolar disorders, lithium is the only mood stabilizer approved by the FDA for acute mania and maintenance treatment [11]. In a recent meta-analysis of studies on the effects of lithium on thyroid function, the investigators compared the prevalence of hypothyroidism in patients treated with lithium with controls. The overall odds of clinical hypothyroidism were close to six times greater in lithium-treated subjects than in controls. Conversely, this study did not find any difference between patients and controls on lithium with respect to the prevalence of increased thyroid function [4]. On the contrary, lithium-induced hyperthyroidism is rare even in adults [12]; it is estimated to have the annual incidence of 0.1% [13]. Other studies have estimated the prevalence to be in the range of 1%–1.7% [5, 14]. Only a small number of children treated with lithium present with subclinical or clinical hypothyroidism, and/or goiter [15, 16], to our knowledge, ours is the first case of a pediatric patient with lithium-induced hyperthyroidism. The possible mechanism regarding lithium-induced hyperthyroidism is thought to be due to an expansion of the intrathyroidal iodine pool, induction of autoimmune hyperthyroidism (Graves' disease), and a toxic thyroid nodule or painless thyroiditis [7, 17]. In patients with primary affective disorders, thyroid autoantibodies were detected in 8 of 40 subjects treated with lithium, and only in 3 patients were treated with other psychotropic drugs. Furthermore, the lithium-treated patients exhibited significantly reduced numbers of suppressor and or cytotoxic T cells and increased B cell activity compared to those treated with drugs other than lithium [18]. Similar to amiodarone-induced toxic thyroiditis, painless thyroiditis may also be the result of a direct toxic effect of lithium on the thyroid, which seems to be the likely mechanism in our patient, in whom results of tests for thyroid antibodies remained negative. Furthermore, it has been reported that lithium-associated silent thyroiditis occurred with an incidence rate of approximately 1.3 cases per 1000 person-years, and lithium-associated thyrotoxicosis occurred with an incidence rate of approximately 2.7 cases per 1000 person-years, higher than the reported incidence rates of silent thyroiditis (<0.03–0.28 cases per 1000 person-years) and of thyrotoxicosis (0.8–1.2 cases per 1000 person-years) in the general population and calculated the odds of lithium exposure to be 4.7-fold higher in patients with thyroiditis [7]. Recently, a meta-analysis of 52 studies showed in nine of these that were cross-sectional or cohort studies describing around 2500 patients that 2.6% patients were hypothyroid [19]. Another cross-sectional study reported just one patient (1.2%) in their lithium-treated group [20]. The histopathological features of the thyroid gland showed an immunological phenomenon with lymphoid follicles and fibrosis in affected thyroid glands [21], and this autoimmune process was not detected in other prospective studies, pre- and postlithium treatment [22, 23]. In our patient, the thyroid antibodies were negative; CBC, C-reactive protein (CRP), and sedimentation rate (ESR) were normal, suggesting that a mild silent thyroiditis may have been the cause of hyperthyroidism. Miller and Daniels described that most hyperthyroid patients had serum lithium level in the therapeutic range [7]. However, our patient's lithium level was not elevated due to discontinuation of treatment 2 weeks before the levels were checked. The duration of lithium therapy in patients who develop hyperthyroidism and lithium-associated silent thyroiditis range between 6 days and 15 years. However, in some cases, thyrotoxicosis occurred 4 days to 5 months after withdrawal of lithium therapy [24], and in our patient, the duration of lithium therapy was about 2 years. In conclusion, lithium-induced hyperthyroidism is very rarely diagnosed in adults and has never been reported in children before our case. We believe it is necessary to increase awareness of the possibility of such drug-induced thyroid dysfunction in the pediatric population, especially in children with hyperthyroidism and negative thyroid autoantibodies. The transient nature of this disorder is associated with a better prognosis compared to patients with Graves' disease. Since lithium-induced hyperthyroidism may occur even after years of lithium therapy, it is advisable to regularly monitor the thyroid function in children during the whole duration of lithium treatment.

## Figures and Tables

**Table 1:** Hormonal profile of the patient with gradual improvements of thyroid hormone levels from being hyperthyroid to euthyroid.

Timeline	TSH (0.35–5.5 mcIU/mL)	Free T4 (0.9–1.9 ng/dL)	Total T3 (55–209 ng/dL)
Initial lab work	0.01	3.5	
2 days later	<0.02	3.7	351
2 weeks later	<0.02	2.0	
1 month later	<0.02	1.5	
2 months	<0.02	0.9	188
3 months	0.39	0.8	175
5 months	2.61	1.2	172
7 months	2.62	1.1	134
